# The Kinetoplast of Trypanosomatids: From Early Studies of Electron Microscopy to Recent Advances in Atomic Force Microscopy

**DOI:** 10.1155/2018/9603051

**Published:** 2018-06-19

**Authors:** Danielle Pereira Cavalcanti, Wanderley de Souza

**Affiliations:** ^1^Laboratório de Microbiologia, Diretoria de Metrologia Aplicada às Ciências da Vida, Instituto Nacional de Metrologia, Qualidade e Tecnologia-Inmetro, Rio de Janeiro, RJ, Brazil; ^2^Instituto Nacional de Ciência e Tecnologia de Biologia Estrutural e Bioimagem and Centro Nacional de Biologia Estrutural e Bioimagem (CENABIO), Universidade Federal do Rio de Janeiro, Rio de Janeiro, RJ, Brazil; ^3^Laboratório de Ultraestrutura Celular Hertha Meyer, Instituto de Biofísica Carlos Chagas Filho, Universidade Federal do Rio de Janeiro, Rio de Janeiro, RJ, Brazil

## Abstract

The kinetoplast is a specialized region of the mitochondria of trypanosomatids that harbors the most complex and unusual mitochondrial DNA found in nature. Kinetoplast DNA (kDNA) is composed of thousands of circular molecules topologically interlocked to form a single network. Two types of DNA circles are present in the kinetoplast: minicircles (0.5–10 kb) and maxicircles (20–40 kb). Knowledge of kinetoplast architecture is crucial to understanding the replication and segregation of kDNA circles because the molecules involved in these processes are precisely positioned in functional domains throughout the kinetoplast. The fine structure of the kinetoplast was revealed in early electron microscopy (EM) studies. However, an understanding of the topological organization of kDNA was only demonstrated after the development of protocols to separate kDNA from nuclear DNA, followed by EM observations. Electron microscopy analysis of thin sections of trypanosomatids, spreading of isolated kDNA networks onto EM grids, deep-etching studies, and cytochemical and immunocytochemical approaches are examples of techniques that were useful for elucidating the structure and replication of the kinetoplast. Recently, atomic force microscopy has joined this set of techniques and improved our knowledge about the kDNA network and revealed new details about kDNA topology in trypanosomatids.

## 1. Introduction

The kinetoplast is a diagnostic structure of the Kinetoplastida order, which encompasses the Trypanosomatidae family. This family of flagellate protozoa comprises species of several genera (*Crithidia*, *Angomonas*, *Strigomonas*, *Trypanosoma*, *Leishmania*, and others). Some trypanosomatids cause tropical human illness such as leishmaniasis, Chagas disease, and African sleeping sickness [1]. Kinetoplastids also include biflagellate free-living protozoa of the suborder Bodonina [2, 3]. Due to the medical importance of trypanosomatids, they have been extensively investigated, and most of our knowledge about kinetoplast comes from this group.

**Figure 1:**
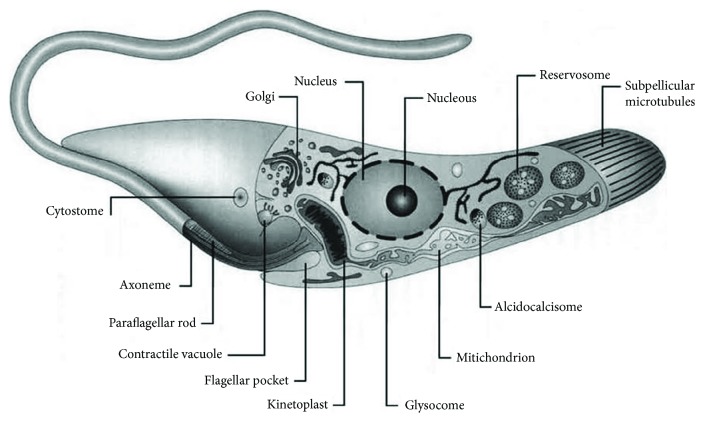
Schematic view of the primary structures and organelles found in the trypanosomatid *T. cruzi*. Reproduced from [18].

The first evidence of the existence of kinetoplast came over a century ago after the discovery of a basophilic granule located near the base of trypanosome's flagellum. However, only after the advent of the transmission electron microscope (TEM) was it possible to understand the structure and nature of kinetoplast [4, 5]. The kinetoplast of trypanosomatids is a specialized region of the mitochondria that harbors the most complex and unusual mitochondrial DNA found in nature (Figure 1). This structure is located near the basal body of the flagellum, and the position of the kinetoplast–flagellum relative to the nucleus is an important feature to classify the different life cycle stages of these protozoa [6]. The kinetoplast DNA, or simply kDNA, represents about 30% of the total cellular DNA and consists of thousands of circular molecules, which are topologically interlocked to form a single network. Two types of DNA rings are present in the kinetoplast lumen: minicircles typically ranging in size from 0.5 to 10 kb (depending on the species) and maxicircles varying in size from 20 to 40 kb [7, 8]. Maxicircles are present in few identical copies per network and are analogous to the mitochondrial DNA of higher eukaryotes; they encode rRNAs and proteins of the respiratory chain. Minicircles are present in numerous copies per network, are heterogeneous in nucleotide sequence, and encode guide RNAs, which are used in the RNA editing process. This process consists of the addition or removal of uridylated residues of the maxicircle transcripts to form functional RNAs [9].

**Figure 2:**
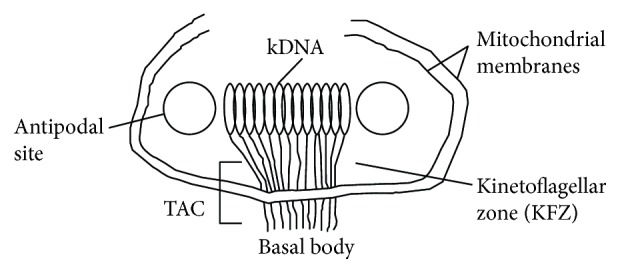
Schematic view of the domains found in the kinetoplast of trypanosomatids. During replication, the minicircles are released from the network into the kinetoflagellar zone, which corresponds to the region between the kDNA and the basal body and contains proteins that initiate the replication of the minicircles. The next steps of replication occur at the antipodal sites, which also contain proteins involved in kDNA duplication. The maxicircles remain attached to the network during their replication, and the details of this process are less well understood.

The replication of kDNA is a complex mechanism that involves a repertoire of proteins and culminates in the segregation of minicircles and maxicircles for daughter cells. The accurate segregation prevents the loss of essential minicircles, without which the edition of maxicircles could not occur and the cells would lose viability [10]. kDNA synthesis involves the release of individual minicircles from the network by a topoisomerase II enzyme, followed by the replication of them as free individual circles. Minicircle replication initiates at conserved origin sequences that are bound by universal minicircle sequence-binding proteins (UMSBP). Abu-Elneel et al. demonstrated that these proteins are located in two neighboring sites adjacent to the face of the kDNA disk closest to the cell flagellum [11, 12]. After this, the replicating minicircles move to the two antipodal sites that flank the kDNA disk, where most but not all primers are removed and the gaps between Okazaki fragments are repaired. Minicircles containing at least one gap are then reattached to the network by topoisomerase II in the antipodal sites, where final gap filling and sealing occur, prior to the network scission and segregation [12]. In contrast, the maxicircles remain attached to the network during their replication in a mechanism involving rolling circle intermediates [13]. The architecture of the kinetoplast is crucial for replication and segregation of the kDNA circles because the molecules involved in these processes are precisely positioned in functional domains throughout the kinetoplast (Figure 2). The kinetoflagellar zone and the antipodal sites are domains involved in the early and late steps of kDNA replication, respectively. In addition, the tripartite attachment complex (TAC), which connects the kDNA to the basal body, mediates the kinetoplast segregation following kDNA replication [14, 15]. For more details about kDNA replication, we refer the interested reader to the excellent reviews published by the groups of Shapiro and Englund, Jensen and Englund, Liu et al., and Klingbeil and Englund [7, 8, 16, 17].

Transmission electron microscopy and more recently atomic force microscopy (AFM) are powerful tools for revealing kinetoplast structure, the topological organization of kDNA, and the replication and segregation of kDNA of trypanosomatids. In this review, we report a range of microscopy techniques that have helped to unravel the intriguing structure of kDNA, considering the initial studies using TEM until the recent advances using atomic force microscopy.

## 2. Visualization of Kinetoplast in Thin Sections

Observations of thin sections of resin-embedded samples have enabled the investigation of the cell components of trypanosomatids, including kinetoplast. Using TEM, Meyer and coworkers [4] were pioneers in demonstrating that the kinetoplast (initially called the kinetonucleus) was separated from the basal body, from which the flagellum emerges. Meyer and colleagues described the kinetoplast as a vacuole-like space containing an electron-dense mass that is now known to be the kDNA. Additionally, this “vacuole” was in direct continuation with the large canal system observed in the cytoplasm of trypanosome, which exhibits in some preparations a lamellar structure that has been described as typical for mitochondria in tissue cells [4, 18]. What Meyer and coworkers described at that time has been confirmed by Paulin [19]. Using serial thick-sectioning techniques combined with electron microscopy (EM), Paulin demonstrated a unique and highly ramified mitochondria in trypanosomatids, with the kinetoplast being part of this structure.

The observation of transversal sections of kinetoplast in various trypanosomatids revealed that this structure is surrounded by the double membrane of mitochondria, from which cristae can occasionally be seen projecting into the lumen. Within the lumen, the kDNA is organized as a densely packed mass of DNA, which is always located perpendicular to the longitudinal axis of the flagellum. Furthermore, the kDNA strands are aligned parallel to the axis of the network. The EM images obtained by Ogbadoyi and coworkers [14] were useful for characterizing the set of filaments that connects the kinetoplast to the basal body of the flagellum. These components comprise a structure that was named the tripartite attachment complex. The physical linkage between the kinetoplast and the basal body had already been demonstrated by Souto-Padrón et al. [20], as will be discussed in the next section. The existence of a physical connection between these two structures explains the early light microscopy observations showing a close relation between them.

**Figure 3:**
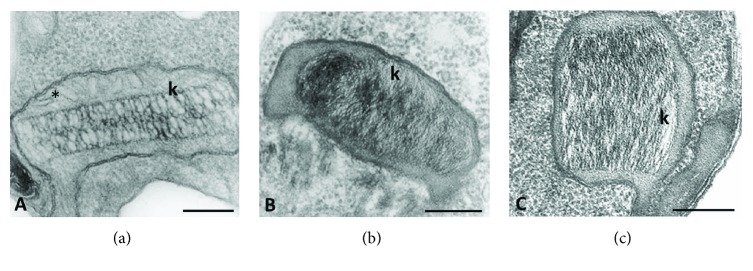
Electron microscopy images of kinetoplasts of nonpathogenic trypanosomatids. The disk-shaped kinetoplast of *C. fasciculata* can be observed in (a), along with the mitochondrial cristae in the kinetoplast lumen (^∗^). The typical kDNA arrangement of the endosymbiont-bearing species *S. culicis* and *A. deanei* can be observed in (b) and (c), respectively. k = kinetoplast. The scale bar corresponds to 0.25 *μ*m. (a) and (b) were reproduced from [53], and (c) was reproduced from [24].

**Figure 4:**
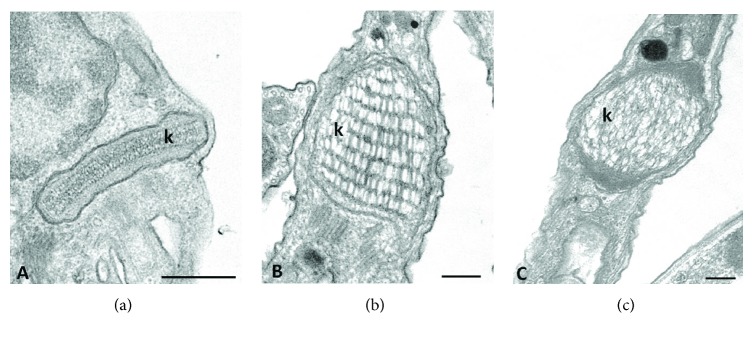
Electron microscopy images of kinetoplast from the parasitic protozoan *T. cruzi*. The kinetoplast of epimastigote form is a disk-like structure containing densely packed DNA fibers (a). When the parasite differentiates and turns into a trypomastigote, an increase in the relative volume of the kinetoplast can be observed, as well as the change in the kDNA topology (b, c). In trypomastigotes, the kDNA fibers are loosely arranged into a globular structure and can be organized in well-defined multilayers (b) or in an irregular arrangement without identifiable layers (c). k = kinetoplast. The scale bar corresponds to 0.5 *μ*m.

The kinetoplast varies in size and kDNA arrangement among species. For example, in *Trypanosoma brucei*, the entire kinetoplast appears as a slightly concave disk about 0.6 *μ*m in diameter and 0.1 *μ*m thick. In epimastigotes of *Trypanosoma cruzi*, the disk has a diameter of 1 *μ*m and a thickness of 0.1 *μ*m. The difference in kDNA arrangements of trypanosomatid species is clearly visible under EM [5]. In the majority of protozoa, the kDNA is tightly packed into a disk-shaped structure (Figures 3(a) and 4(a)). However, in species that harbor an endosymbiotic bacterium, the kinetoplast presents a trapezoid or arch-shaped structure completely filled with loosely packed kDNA strands (Figures 3(b) and 3(c)) [21]. The kinetoplast of trypomastigote of *T. cruzi* is globular and can present two types of topologies: one consisting of well-defined multilayers and the other consisting of an irregular arrangement without identifiable layers (Figures 4(b) and 4(c)) [22].

## 3. Three-Dimensional View of the Kinetoplast Revealed by Deep Etching

**Figure 5:**
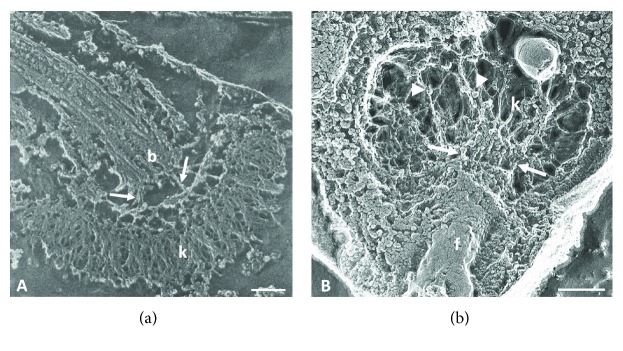
Deep-etching analysis of the kinetoplast of *T. cruzi* (a) and *A. deanei* (b). The images show the arrangement of kDNA fibers as well as the filaments connecting the kDNA to the flagellum (arrows). In the kinetoplast of *A. deanei*, the presence of globular structures is indicated by arrowheads. b = basal body; f = flagellum; k = kinetoplast. The scale bar corresponds to 0.25 *μ*m. (a) was reproduced from [20], and (b) was reproduced from [24].

A three-dimensional view of kDNA filaments could be obtained observing replicas of quick-frozen, freeze-fractured, and deeply etched trypanosomatids. In this methodology, the sample is exposed to rapid cryofixation that prevents the retraction and redistribution of kDNA fibrils. The deep-etching technique was applied to *T. cruzi* in the early 1980s, enabling observation of the kDNA organization in situ. The deep-etching images of Souto-Padrón et al. [20] revealed that the kDNA filaments were packaged in dense bundles within the kinetoplast of *T. cruzi* epimastigote and that filaments roughly 11 nm in diameter connected the kDNA to the basal body (Figure 5(a)). Until then, no previous ultrastructural studies had demonstrated a structural linkage between these two structures, although it was known that the kinetoplast and flagellum remained together during isolation [23]. Deep-etching analysis also provided a three-dimensional view of the kinetoplast of the endosymbiont-bearing trypanosomatid *Angomonas deanei*, as presented by Cavalcanti et al. [24]. This technique revealed that the typical kDNA network of this protozoan was made up of DNA filaments arranged in a less-condensed manner than that of *T. cruzi* kDNA and occupying the entire kinetoplast matrix (Figure 5(b)). This species also presented filamentous structures linking the kDNA to the basal body, which likely corresponds to the filaments of the TAC. Furthermore, the deep-etching images of *A. deanei* demonstrated the presence of globular structures throughout the kDNA network that may correspond to the proteins involved in the kDNA compaction (Figure 5(b), arrowheads).

Although the observation of thin sections and deep-etching replicas has yielded important contributions to studies of kinetoplast, the highly complex organization of the kDNA network can be better understood only after its isolation and observation, initially under TEM, and more recently using AFM.

## 4. Investigating the Topology of Kinetoplast DNA: Early Observation in EM Grids

A better understanding of the topological organization of the kDNA was possible due to the development of protocols to separate kDNA from nuclear DNA using density-gradient centrifugation technique. Several studies published in the 1960s and the 1970s characterized the kDNA molecules isolated from *Leishmania enriettii* [25], *T. cruzi* [26, 27], *Trypanosoma mega* [28], *Leishmania tarentolae* [29, 30], *Crithidia* species [31], *Crithidia fasciculata* [32], and other trypanosomatids. These studies promoted the knowledge that we have today about the remarkable molecular configuration of kDNA. The EM analysis of isolated kDNA spread in microscopic grids and shadowed with metals revealed that the predominant structural element of kDNA was circular molecules whose contour lengths varied among species. For example, in *T. cruzi*, the circles are 0.45 *μ*m long, corresponding to 1440 base pairs and a molecular weight of 0.94 × 10^6^ daltons [27]. The contour length of circles in *L. tarentolae*, *T. mega*, and *C. fasciculata* are 0.29, 0.74, and 0.8 *μ*m, respectively [28, 29, 32]. Simpson and Da Silva [29] proposed that the diameter of the minicircle corresponds to the width of the kinetoplast observed in thin sections of resin-embedded cells. Therefore, the kinetoplast ultrastructure would be determined by the size of the minicircle formed by a single minicircle tier in most of the trypanosomatids studied.

Although long linear DNA was repeatedly observed in EM micrographs of isolated kDNA of *T. cruzi* and *Leishmania*, conclusive evidence of the presence of a second structural element in the kDNA network was only obtained by Steinert and Assel [33]. Electron microscopy analysis of kDNA isolated from *Crithidia luciliae* and restriction endonuclease digestion assays led to the identification of kDNA molecules larger than the minicircles, and these long molecules were called “maxicircles” [33, 34]. Based on results obtained after treating the kDNA network with specific endonucleases, Kleisen and coworkers [34] demonstrated that the maxicircles were a constant and integral part of intact networks of *C. luciliae* and represented 3–5% of the mass of the kDNA structure. In addition, the authors suggested that maxicircles were equivalent to the mitochondrial DNA in other organisms.

**Figure 6:**
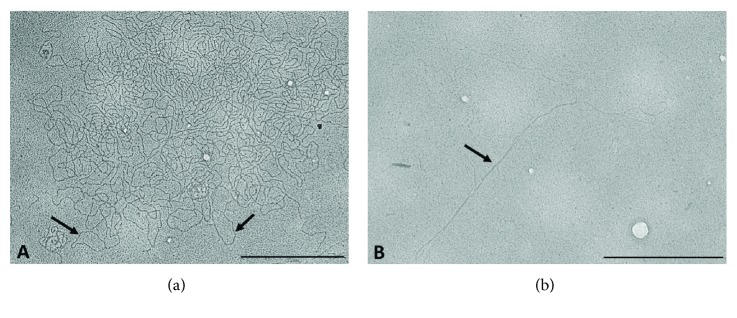
Electron microscopy image of the components of the kDNA network of epimastigote of *T. cruzi*. Minicircles isolated from the network are indicated by the arrows (a). The maxicircle is indicated by the arrow in (b). The scale bars correspond to 0.5 *μ*m.

The isolated kDNA network is a planar structure containing thousands of minicircles and dozens of maxicircles interlocked in an unusual arrangement (Figure 6). The minicircles are organized in the form of rosettes, which have a center from which DNA loops radiate. The rosettes are connected to one another by bundles of filament. The maxicircles, on the other hand, form independent catenanes within kDNA networks that are linked to one another within the catenane of the minicircles [35].

Because kDNA networks have a diameter comparable to that of the entire cell, an efficient mechanism of kDNA condensation must exist so that the network fits within the mitochondrial lumen. Small, highly basic kinetoplast-associated proteins (KAPs) are candidates for packaging and organizing the kDNA [36, 37].

Recently, the topological organization of the kDNA was revisited by our group using AFM. The challenges and advantages of using AFM to study the kDNA will be discussed in the next section.

## 5. Atomic Force Microscopy as a Tool to Investigate Kinetoplast DNA Organization: Advantages and Challenges

The advent of AFM in 1986 opened up an exciting perspective for the study of biological specimens [38]. AFM has several features that render it attractive to biologists: it covers a wide range of resolution (from nm to *μ*m); it can be used for imaging at atmospheric pressure; and the sample preparation for AFM imaging is normally very fast and simple, as there is no need for stains, shadows, labels, or other procedures that could create artifacts in the material. In addition, an important advantage of AFM over EM is its ability to measure heights and therefore obtain topographic maps of cells and nanometer samples such as proteins and nucleic acids. Early publications related to observations of the DNA structure using AFM included imaging of single- and double-stranded DNA/RNA molecules [39–41], linear and circular DNA [42, 43], relaxed and supercoiled plasmids [44, 45], and the kDNA network of the trypanosomatid *C. fasciculata* [46]. The preparation of kDNA samples for AFM analysis was initially a major challenge because thousands of interlocked DNA molecules had to be completely scattered on a surface to be observed. In addition, since AFM uses a probe to scan the surface, the large kDNA network must be firmly attached to the substrate so that the sample will not be dragged by the tip. Prior knowledge related to immobilizing linear DNA and plasmid samples for AFM was useful for working with kDNA. However, the methods described to dry small DNA molecules for AFM observation were not successful with the kDNA network, as discussed below.

The first studies to prepare nucleic acids for AFM assays were focused on obtaining reliable and reproducible protocols to adsorb these molecules in a substrate without changing their structures. Satisfactory results were obtained when the DNA-cytochrome C copolymer was deposited on carbon-coated mica in a procedure similar to that used to spread DNA on microscopy grids [44, 47]. Chemical modifications of mica to increase the binding of DNA to the substrates were also used, as well as cation-assisted procedures. These cation-assisted procedures consist of pretreating the mica surface with divalent cations or using divalent cations (such as magnesium) directly in the DNA solution prior to depositing the sample onto the mica. The latter technique is widely used due its simplicity and reproducibility. In both cases, it is believed that the cations act as bridges between the negatively charged mica and the negatively charged DNA [48, 49]. The addition of magnesium in the kDNA solution, followed by adsorption of the sample in the mica and rinsing steps to remove the excess buffer salt, was successful at obtaining high-resolution AFM images of trypanosomatids, as reported by Cavalcanti et al. [50].

**Figure 7:**
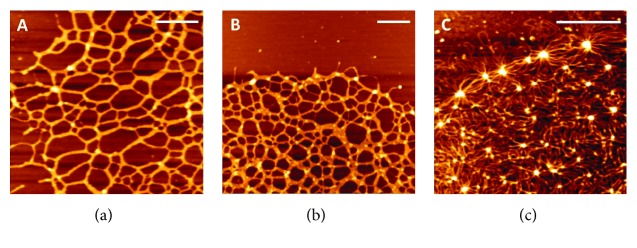
Atomic force microscopy images of the kDNA network of *C. fasciculata* dried under vacuum (a), using a nitrogen flow (b) or critical point drying (c). The sample was adsorbed onto freshly cleaved mica using a MgCl_2_ buffer solution, rinsed gently in Milli-Q water, and dried. The images were acquired by a NanoWizard AFM (JPK Instruments AG, Germany) in air using the intermittent mode. The scale bars correspond to 0.5 *μ*m. Figure adapted from [49].

In addition to immobilizing the material on a surface, drying the sample is also a critical step for preparing biological specimens for AFM. The drying procedure can drastically change the physical properties of DNA, such as the height and diameter of the molecule. The height and diameter of the kDNA samples submitted to drying with nitrogen flow or a vacuum chamber were larger than those of samples dried using the critical point technique. For example, the diameter of kDNA molecules was roughly 40, 33, and 16 nm for samples dried under vacuum, nitrogen flow, and critical point drying (CPD), respectively. Furthermore, the first two protocols promoted coalescence of kDNA strands and breakage of the kDNA molecules located at the periphery of the network (Figures 7(a) and 7(b)) because the drying forces compressed the strands of kDNA [49]. To overcome this problem, CPD, which has been extensively used for preparing highly hydrated biological material for scanning EM, was our method of choice for preparing the kDNA network for AFM. In this technique, the sample is dehydrated in a solvent such as ethanol and later the solvent is replaced by liquid carbon dioxide in the pressure chamber of the CPD device. When the temperature and pressure exceed the so-called critical point of carbon dioxide (31°C and 74 bar), the pressure is carefully reduced and the sample is removed once atmospheric pressure is attained. Therefore, the sample is dried without ever having crossed the liquid-gas phase border, which can majorly distort biological specimens. High-resolution images of the kDNA network were obtained using the CPD technique, which made it possible to examine the DNA strands in detail (Figure 7(c)).

**Figure 8:**
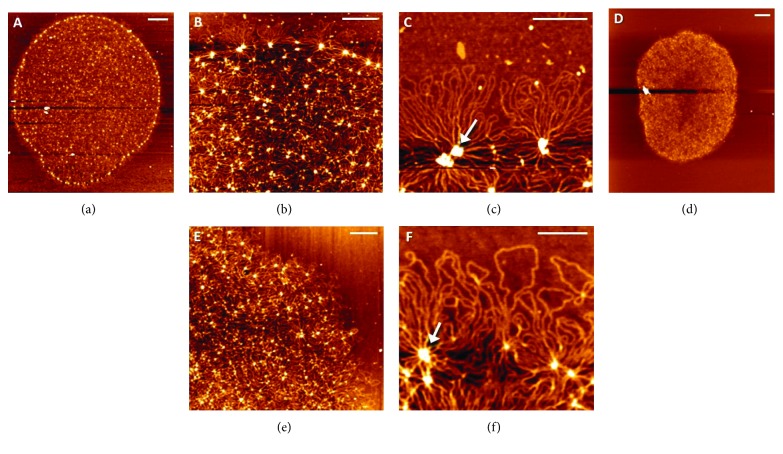
Atomic force microscopy analysis of the isolated kDNA network of trypanosomatids. (a–c) Network of *C. fasciculata* and (d–f) network of *A. deanei.* The entire networks are shown in (a) and (d). Details of the networks are shown in (b), (c), (e), and (f). (c, f) Clustering of DNA molecules forming rosettes is observed in the periphery of the network (arrows). The scale bars correspond to 1 *μ*m (a, d), 0.5 *μ*m (b, e), or 0.25 *μ*m (c, f). (a), (b), and (c) reproduced from [50].

Atomic force microscopy has been used to observe the kDNA topology of trypanosomatids such as *C. fasciculata* [50], *A. deanei*, *Strigomonas culicis* [21], and *T. cruzi* [22]. All of the species exhibited similar sizes of kDNA (Table 1) and appeared as intact and massive networks with a high concentration of DNA fibers distributed throughout their structure. Clusters of DNA molecules forming rosettes were noted along the networks in all of the trypanosomatids studied (Figure 8). The center of each rosette knot appeared to be connected to other rosette knots via bundles of filaments, which organized the intricate structure of the kDNA network (Figure 8, arrows). The new information provided by AFM analyses compared to electron microscopy studies was related to the measurement of kDNA height at different sites in the network. The height measurement allowed us to calculate the amount of overlapping circles at various points in the kDNA network, especially at sites where rosette-like structures were formed. For example, in *C. fasciculata*, the height of an individual kDNA molecule was 0.5 nm. However, when the entire network was analyzed, the height of kDNA varied from 0.5 nm to 10.5 nm. The highest points correspond to the sites in which several kDNA molecules intersect, forming the nodes of each rosette. This height variation indicated that the rosettes comprise up to 20 molecules of kDNA that converge and cross over themselves. It is interesting to point out that in the middle of the network, the height varied from 0.5 to 3.5 nm while in the periphery, it varied from 0.5 to 10.5 nm. These data demonstrated that the rosettes at the periphery of the network had a greater number of DNA molecules superimposed than the central region [50]. Similar results were obtained when kDNA networks of *A. deanei* and *S. culicis* were analyzed [21].

Observing the data of Table 1, we note that the kDNA isolated from distinct trypanosomatids exhibited a very similar pattern of organization and sizes (Figure 8). However, these networks occupy kinetoplasts of different shapes and dimensions. These data suggest that the densely packed arrangement of kDNA fibers within the three-dimensional structure of the kinetoplast is an event driven by a spatial condition rather than being caused by a distinct bidimensional structural organization of kDNA molecules among trypanosomatids. Moreover, the interaction of kDNA with numerous proteins must also contribute to maintaining its ultrastructural organization.

**Figure 9:**
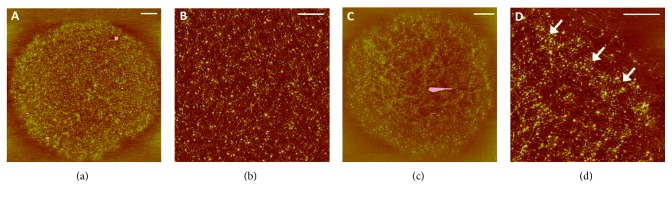
Atomic force microscopy analysis of the isolated kDNA network of epimastigote (a, b) and trypomastigote (c, d) of *T. cruzi*. The kDNA is composed of a massive network of interlocked DNA molecules. The kDNA molecules are uniformly distributed in epimastigote (a, b) while in trypomastigote (c, d) several foci containing high concentrations of kDNA fibrils were observed (d, arrows). The scale bars correspond to 1 *μ*m (a), 2 *μ*m (c), or 0.5 *μ*m (b, d). Figures adapted from [22].

Recently, the AFM was used to investigate the arrangement of kDNA in distinct life cycle stages of *T. cruzi*, the protozoan that causes Chagas disease. During its life cycle, this parasite undergoes major changes in the kinetoplast morphology and kDNA topology as it passes from the epimastigote replicative stage in the insect vector to the metacyclic trypomastigote form, which infects humans, in a process known as metacyclogenesis. The comparative analyses of epimastigote and trypomastigote highlighted interesting differences in the topology of kDNA. While in epimastigote the kDNA fibers presented a uniform distribution along the network, in trypomastigote a large number of sites containing a higher concentration of kDNA fibers was observed (Figure 9, arrows). The regions with higher concentration of DNA presented higher height, indicating that there was agglomeration of molecules in these sites. This result suggests that the kDNA undergoes compactation during differentiation from epimastigote to trypomastigote and can explain the smaller size of the network observed in trypomastigote (Table 1). The biological significance of the changes in kDNA topology during differentiation of *T. cruzi* is not fully understood, but may be the result of a generalized mechanism for gene expression repression in the kinetoplast DNA [22]. It is important to emphasize that metacyclogenesis is an adaptive differentiation that enables *T. cruzi* to survive in diverse environments. During differentiation, the parasite takes on distinct forms as result of changes in gene expression that lead to morphological, ultrastructural, metabolic, and physiological modifications.

Atomic force microscopy has been successfully applied not only to evaluate kDNA structure but also to investigate changes in kDNA topology caused by drugs. It has been reported that kDNA constitutes a potent target for chemotherapy because it is strongly affected by DNA-binding drugs, intercalating agents, and topoisomerase inhibitors that interfere with its unique structure and replication. Our group analyzed the effect of acriflavine, an intercalating drug, and berenil, a minor-groove binding agent, on the *T. cruzi* kDNA network using AFM [51, 52]. Our results shed light on how these drugs affect the kDNA organization and contribute to parasite death. The use of AFM to analyze the effect of compounds affecting the kDNA network has the advantage of easy preparation of the material, without the use of stains, shadows, labels, or other procedures that could introduce artifacts into the sample and mask the effect of drugs. The study of the action of acriflavine on *T. cruzi* was a successful example of the use of AFM to improve understanding of how this drug disrupts kDNA. When used in low concentration, acriflavine caused the release of kDNA circles from the periphery of the kDNA network. However, when used in higher concentration, the drug promoted detachment of network bulges, resulting in kDNA fragmentation and the phenomenon of diskynetoplasty early described in EM analyses [51]. The use of AFM allowed a detailed monitoring of the release of the circles from the network in the initial stages of acriflavine action until the total dismantling of the network caused by the drug.

Taken together, the data presented here demonstrated that AFM is an excellent tool for analyzing the disruption of the kDNA network caused by drugs, as well as to evaluate the topology of kDNA networks, complementing the studies of electron microscopy. In addition, new applications could be obtained performing AFM imaging in liquid or using AFM to investigate the interaction of proteins with specific regions of minicircles and maxicircles.

## 6. Conclusion

Atomic force microscopy has been successfully applied to study the structure of nucleic acids, DNA-protein interactions, and damage to DNA structure caused by drug or radiation exposure. In recent years, AFM has been used to study kDNA topology and the effect of drugs on the kinetoplasts of trypanosomatids. The combination of EM techniques and AFM can improve our knowledge about kinetoplast and reveal new details about the intriguing structure of kDNA.

## Figures and Tables

**Table 1:** Measurements of the kDNA network of some trypanosomatids.

Trypanosomatids	kDNA network sizes (*μ*m)	Height	Ref.
*Crithidia fasciculata*	7.72 × 6.89 ± 0.64 × 0.65	0.5 to 10.5 nm	[50]
*Angomonas deanei*	7.34 × 6.47 ± 0.10 × 0.30	0.4 to 8.0 nm	[21]
*Strigomonas culicis*	7.43 × 6.34 ± 1.10 × 0.80	0.5 to 8.0 nm	[21]
*T. cruzi* (epimastigote)	9.54 × 8.62 ± 1.09 × 1.20	ND	[22]
*T. cruzi* (trypomastigote)	7.48 × 6.93 ± 0.66 × 0.58	ND	[22]

ND: not determined by the authors.

## Abbreviations

AFM: Atomic force microscopy
EM: Electron microscopy
kDNA: Kinetoplast DNA
TAC: Tripartite attachment complex
TEM: Transmission electron microscope.
